# Children engage neural reward structures for creative musical improvisation

**DOI:** 10.1038/s41598-025-95619-1

**Published:** 2025-04-10

**Authors:** Karen Chan Barrett, Patpong Jiradejvong, Lauren Jacobs, Charles J. Limb

**Affiliations:** 1 Sound and Music Perception Lab, Department of Otolaryngology-Head and Neck Surgery, School of Medicine, University of California, San Francisco (UCSF), San Francisco, CA 94143 USA; 2 Institute for Health and Aging, School of Nursing, University of California, San Francisco, San Francisco, CA 94158 USA

**Keywords:** Cognitive neuroscience, Human behaviour

## Abstract

Children spontaneously engage in creative behaviors. However, little is known about the biological underpinnings of creativity in children. We identified neural substrates associated with musical improvisation in children aged 9–11. Participants played a non-ferromagnetic piano keyboard in a functional magnetic resonance imaging (fMRI) scanner using a musical paradigm that required no prior musical experience, in which they played a rote pattern from memory or improvised melodies using those same notes. fMRI analysis of children’s brains during musical improvisation revealed (1) heightened functional connectivity between emotion and reward brain areas and (2) deactivation of auditory, limbic, and parietal structures, particularly the middle temporal gyrus, angular gyrus, precuneus, and cingulate cortex. Importantly, improvisation engaged reward structures more than the control condition. Neural results suggest that children possess nascent creativity networks that form the roots for later adult creativity networks.

**Supplementary Information:**

The online version contains supplementary material available at 10.1038/s41598-025-95619-1.

## Introduction

Children are found spontaneously engaging in a range of creative behaviors. 98% of 5-year-old children score in the highly creative range on a creativity test, compared to only 2% in adulthood^1^. Children possess open and flexible minds, which allows them to display new cognitive strategies^2^ and learn novel physical or social causal relationships faster than adults and adolescents^3^. Children perform better on divergent thinking tasks than adults^4^. However, this view is not without contention^5,6^. Evidence suggests that adults aged 25–30 can outperform younger participants in specific measures of creativity, such as *originality* in verbal divergent thinking^7^. Moreover, creativity in adulthood is thought to stem from extensive training and the gradual acquisition of domain-specific expertise, suggesting that children’s creative works lack the sophistication found in mature creators producing substantive contributions^5^. In short, while children undoubtedly demonstrate creative thinking in domains ranging from music to drawing to construction to gardening^8^, questions remain about the neural and cognitive mechanisms that underpin their particular type of creativity. Why do children instinctively pursue creative behaviors? Are there nascent neural networks that support creativity early in development? This study aims to explore the neural substrates of musical creativity in children with minimal prior training, offering novel insights into the origins of creativity during development. To our knowledge, this experiment is the first to identify neural substrates in children as they perform a real-time creative task.

Improvisation, or activity performed spontaneously without pre-planning, is a fundamental human behavior. Humans improvise whenever they converse without a script or execute a novel gesture. Musical improvisation, specifically, represents a unique and complex process that leads to the impromptu generation of rhythms and melodies in a particular musical context and, therefore, represents a “window into real-time creative processes”^9^. When improvising, musicians generate ideas and execute them on the instrument immediately under significant time constraints. Unlike other creative musical processes, such as composition—where material can be revised and refined over time—musical improvisation requires performers to generate and execute ideas under the immediate time constraints of a live performance. Because of this complexity, research over the last decade has attempted to understand the brain activity underlying musical improvisation as an example of creative behavior. Using functional magnetic resonance imaging (fMRI), experiments performed on adult musicians have shown that improvisation is a distinct biological behavior from playing memorized, pre-learned music^10–13^.

Unlike professional adult musicians, children do not have a lifetime of lived experiences or extensive stored musical knowledge to utilize while improvising musically. Yet, they can engage in musically creative activities, among other creative behaviors. What happens in the developing minds of children as they are being creative? Can we identify the neural basis for *why* children readily engage in creative activities without prior experience or training? Behavioral research has shown that utilizing improvisation as a pedagogical technique (compared to didactic musical instruction) increases creative thinking, musical flexibility, originality, and syntax in young children^14^. Even children too young to be skillful in structured composition (i.e., under 7) can improvise, suggesting that improvisation is an excellent introduction to musical learning^15,16^. For children with little to no musical experience, improvisation may be a musical form of play, a behavior that is innately enjoyable and, hence, rewarding.

Previous neuroimaging experiments in highly trained adults required participants to perform memorized and improvised musical material. In adult professional Classical pianists, improvisation resulted in activations in the right dorsolateral prefrontal cortex (DLPFC) and pre-supplementary motor area (preSMA), dorsal premotor cortex (PMD), and the left posterior superior temporal gyrus (STG)^13^. In a study of professional jazz musicians highly skilled in improvisation, a distinctive pattern of neural activation and deactivation was observed during improvisation in comparison to memorization. Improvisation was associated with widespread deactivation of the dorsolateral prefrontal cortex (i.e., DLPFC) and limbic areas coupled with simultaneous activation of the medial prefrontal cortex (mPFC), superior and middle temporal gyrus (STG and MTG) involved in sensorimotor activity, and the anterior cingulate cortex (ACC)^10^. This study suggested that musical creativity requires the activation of brain areas involved in the neural instantiation of the self and self-generated behaviors (i.e., mPFC), concurrently with the suppression of lateral prefrontal activity (i.e., DLPFC), thought to be involved in conscious self-monitoring and judgment^10^. Experienced musicians often report experiencing a “flow state”—an optimal experience of deep focus, creativity, and enjoyment^17^—when improvising^18–20^. This experiment was among the first to use neural imaging to identify neural correlates of both musical improvisation and potential entry into flow states.

Since these early studies, several neural imaging experiments of investigated musical improvisation in adult participants, often focusing on expert, trained musicians. There has been great variety among these neuroscience experiments with respect to the participants, the improvisation task (e.g., mental improvisation^21–23^, collaborative improvisation^24^, rhythmic vs. melodic improvisation^25^), as well as how the task is implemented (playing a keyboard^10,12,13,26^, pressing a button box^11,27^, vocalizing^21,23^, or mentally improvising^21–23^). Despite these experimental differences, however, it has been found that the mPFC and cingulate cortex, the DLPFC, inferior frontal cortices (e.g., inferior frontal gyrus), dorsal premotor areas (for motor planning and sequencing), cerebellum, and preSMA and SMA areas are associated with improvisation^28–30^. Musical improvisation involves neural substrates across functional networks such as the default mode network (DMN) and executive network (EN). The EN, including the DLPFC and ACC, supports cognitive control, while the DMN, encompassing midline structures like the mPFC, posterior cingulate cortex (PCC), precuneus, inferior parietal lobe (IPL), angular gyrus (AG), and supramarginal gyrus (SMG), facilitates mind-wandering and internally directed thought^31^. Functional connectivity research suggests that during improvisation, the DMN generates ideas, passing them to the salience network, which then channels them to the EN for evaluation and refinement^32,33^. This neural interplay aligns with the dual demands of improvisation—balancing idea generation with evaluation^9^, and generative creativity with cognitive control. Furthermore, creative thinking, although not musical improvisation per se, has been linked to intrinsically motivated, novelty-seeking behavior, thus engaging the brain’s reward system^33,34^.

The neural networks and structures involved in generative creativity and musical improvisation, in particular, have been identified in adults. Children, however, may rely on distinct neural substrates or a subset of these structures to support their creativity. Their “freshness of perspective, break with realism, and ‘easiness’ to disregard social conventions” may underpin their distinctive creative abilities, which are not built on expertise on domain-knowledge (pg. 125 ^48^). Other research suggests that curiosity, sociability, and play support creative learning in children^35^. This study aimed to identify the neural substrates of musical creativity in children using functional MRI during an improvisation task vs. a rote memorized musical task. Musically untrained children engaged in a novel experimental paradigm that required no prior musical training or expertise, in which they either played a repetitive pattern of notes (pentatonic scale, ascending/descending using black keys of the piano only) or improvised spontaneous melodies utilizing the same set of pentatonic scale notes. This paradigm created an improvisation condition with no “wrong” notes or possible mistakes, thereby suspending judgments of right or wrong when playing and encouraging a sense of freedom, exploration, and self-expression.

We hypothesized that children would approach musical improvisation and memorization differently, with improvisation representing a form of creative play not shared by a memorization task. As a result of these fundamentally contrasting approaches to creative and memory-based tasks, we hypothesized that children would demonstrate engagement in reward-processing areas of the brain during musical improvisation. Furthermore, due to the relative underdevelopment of executive control, we hypothesized that children would demonstrate the relative absence of EN activity during musical improvisation compared to memorization.

## Results

### Improvisation is associated with neural deactivation of limbic, parietal, motor, and cognitive control areas

**Fig. 1:**
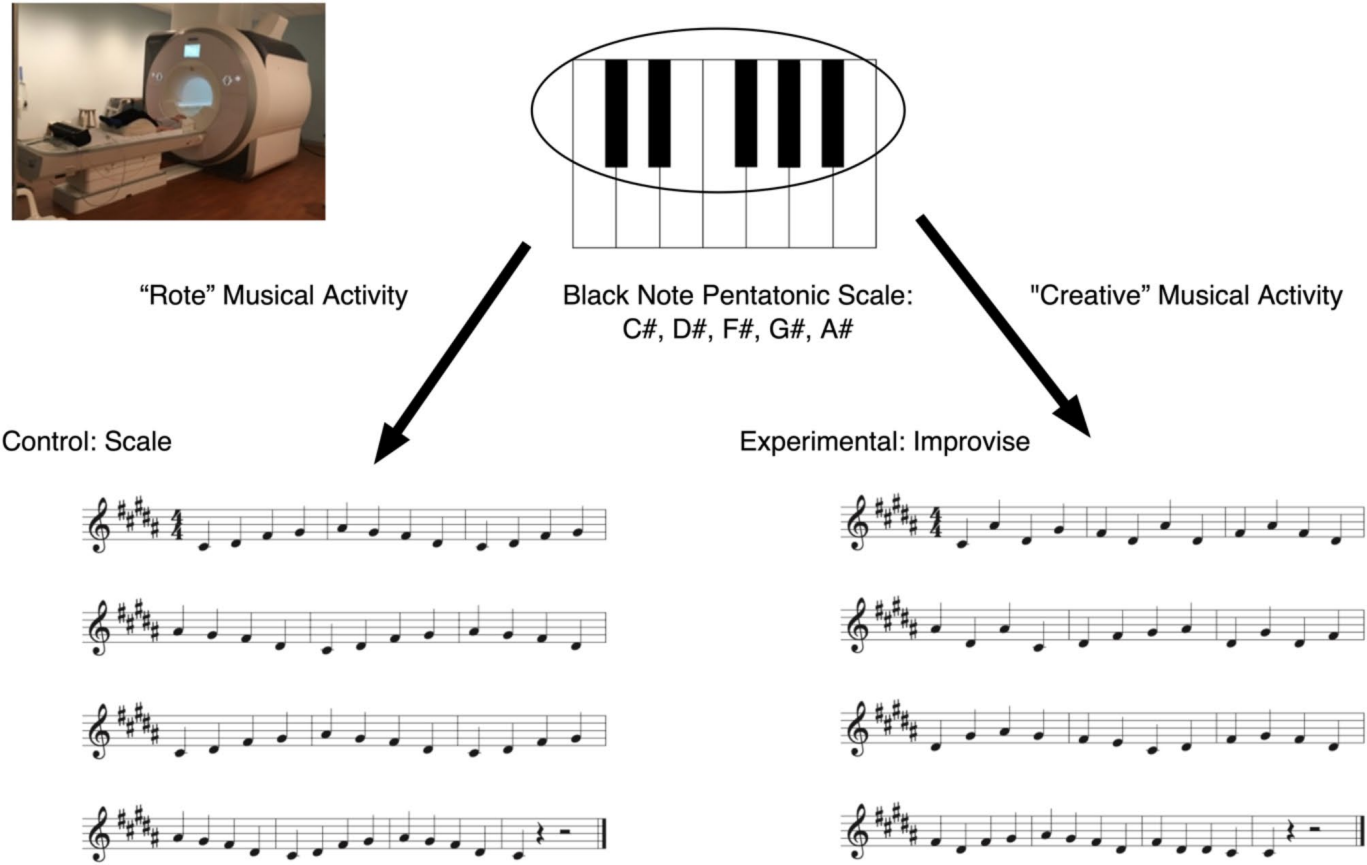
Musical paradigm based on the black note pentatonic scale. A custom-built, non-ferromagnetic MIDI piano keyboard used during fMRI scanning. The keyboard had 35 full-size piano keys, which triggered high-quality piano sound samples generated outside the scanner and immediately routed back to the participants using pneumonic earmuffs. During scanning, participants were randomly cued to play either the control condition or to improvise. Participants repeatedly played the pentatonic scale’s ascending and descending five black note keys in quarter notes for the control condition. For the improvisation condition, participants improvised in quarter notes using only the notes of the pentatonic scale as depicted by musical scores at the bottom of the figure (see supplementary materials for sound files). Inset Picture: Image of child participant lying on fMRI scanner table. The piano is played on the child’s knees inside the scanner bore. Participants can see their hands and the keyboard via the mirror on the scanner head coil.

fMRI scanning featured two runs, each comprising five control blocks and five improvisation blocks presented randomly. Participants lay supine with the custom-made, nonferromagnetic piano keyboard on their knees (Fig. 1). When cued, participants either performed the pentatonic, black note scale up and down repeatedly to the accompanying backtrack, which designated the tempo/speed for the control condition, or created novel melodies using the same notes and played at the same rhythm and speed as the control condition (improvisation, experimental condition). The backtrack was specially curated so that the material always sounded musical; there was no way to make a mistake, encouraging participants to improvise despite having little to no prior musical background.

**Fig. 2:**
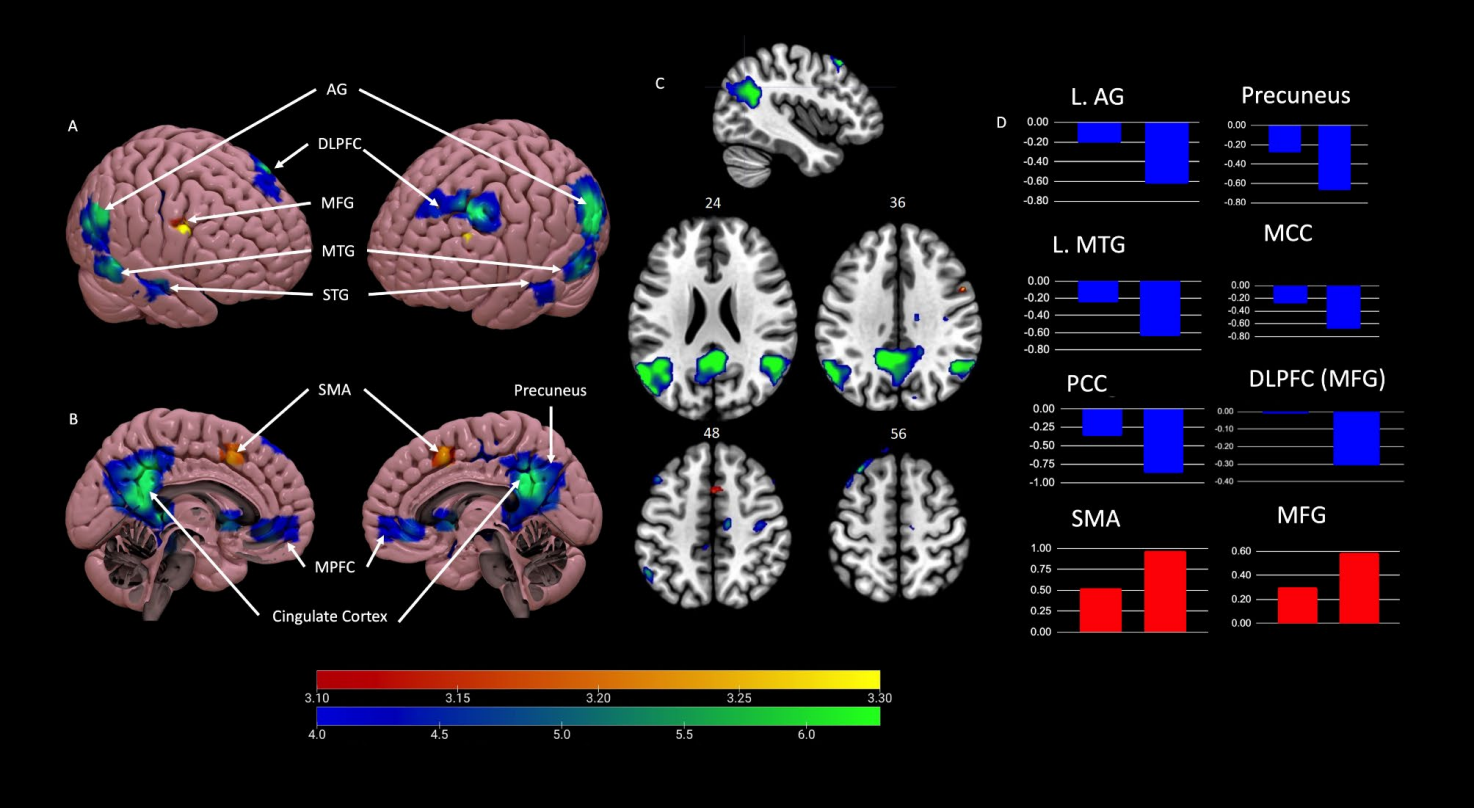
2D and 3D imaging comparing Improvise-Control conditions with neural activation (in red) and deactivation (in blue). Panel (**A**) shows 3D surface projection of data in right and left hemisphere views. Panel (**B**) depicts midbrain views. Panel (**C**) shows 2D axial slices. Improvisation is primarily associated with deactivation of brain areas. Panel (**D**) on the far right shows effect size graphs for key activation (in red) or deactivation clusters (in blue). Bar graphs demonstrate increased neural activation or deactivation (i.e., *F*-score Effect Sizes) in key regions. For each bar graph, each column on the left represents the scale (control condition), while the bar on the right represents improvisation (improvise condition). *L* left, *AG* angular gyrus, *MFG* middle frontal gyrus, *STG* superior temporal gyrus, *MTG* middle temporal gyrus, *MPFC* medial prefrontal cortex, *SMA* supplementary motor area, *MCC* midcingulate cortex, *PCC* posterior cingulate cortex, *DLPFC* dorsolateral prefrontal cortex.

A random effects analysis with masking was used to identify true activation and deactivation clusters involved with musical improvisation. In children, improvisation primarily results in the deactivation of brain structures. Widespread deactivation (Fig. 2) was found in (1) limbic areas such as the posterior cingulate cortex (PCC), midcingulate cortex (MCC), anterior cingulate cortex (ACC) and hippocampus, (2) reward areas like caudate, amygdala, and striatum, (3) auditory temporal areas like the MTG and STG, and (4) parietal sensorimotor areas like the AG, precuneus, and primary motor areas. Smaller focal clusters of deactivation were found in frontal areas like the left posterior DLPFC (BA 8) and mPFC (BA 32). Surprisingly, only a few minimal areas of focal activation were identified: in the bilateral SMA, right premotor area, and left middle frontal gyrus (MFG), part of the DLPFC (see Table 1; Fig. 2). Thus, this fMRI contrast analysis identifies neural structures in children used for generative musical creativity, which consists mainly of deactivation of auditory, limbic, and sensorimotor structures. The most significant clusters of deactivation occurred in the MTG, AG, precuneus, and cingulate cortex (Table 1). Smaller clusters of deactivation occurred in the DLPFC and ACC, structures belonging to the executive network (EN) responsible for cognitive control, thereby supporting our hypothesis that children did not use cognitive control for improvisation. Additionally, deactivation clusters in reward areas indicate that improvisation engages reward structures more than the control condition.

**Table 1:** *Minima* and *Maxima* showing activation (top) and deactivation (bottom) clusters and locations. All coordinates are described according to the Montreal Neurological Institute (MNI) system. *SMA* supplementary motor area, *DLPFC* dorsolateral prefrontal cortex, *MFG* middle frontal gyrus, *mPFC* medial prefrontal cortex, *MTG* middle temporal gyrus, *STG* superior temporal gyrus, *MCC* midcingulate cortex, *PCC* posterior cingulate cortex.

Structure	BA	Left hemisphere					Right hemisphere				
		t-score	x	y	z	Cluster Size	t-score	x	y	z	Cluster Size
**Activations**											
SMA	6	3.32	0	12	48	24	3.32	0	12	48	24
Premotor	6	–	–	–	–	–	3.37	52	6	36	10
DLPFC: MFG	9	3.34	− 40	32	32	33	–	–	–	–	–
**Deactivations**											
Primary motor	4	–	–	–	–	–	4.98	40	− 18	50	105
DLPFC: MFG	6,8	8.71	− 32	28	58	36	6.27	12	− 18	54	57
mPFC	32	4.50	− 2	52	− 4	53	–	–	–	–	–
MTG	21	6.81	− 48	− 58	18	1789	6.44	56	− 18	− 12	241
STG	22	5.55	62	− 4	− 8	241	6.44	56	− 18	− 12	241
Fusiform gyrus	37	5.85	− 20	− 36	− 16	228	–	–	–	–	–
Angular gyrus	39	7.25	− 44	− 54	24	1789	17.39	52	− 54	24	916
Precuneus	31	11.22	− 8	− 46	40	2693	5.87	0	− 64	43	2693
M. occipital gyrus	19	6.76	− 38	− 66	24	1789	–	–	–	–	–
ACC	32	5.68	4	40	− 6	52	5.68	4	40	− 6	52
MCC	31, 24	10.91	− 10	− 48	40	2693	5.74	14	− 14	48	57
PCC	23, 31	11.89	− 2	− 52	24	2693	13.96	2	− 50	28	2693
Hippocampus/parahippocampus	–	7.98	− 26	− 34	− 8	228	4.82	26	− 20	− 12	24
Caudate	–	6.15	− 4	12	0	38	4.34	4	10	0	38
Amygdala	–	–	–	–	–	–	5.27	30	2	− 16	51
Striatum (nucleus accumbens)	–	5.34	− 4	12	− 2	38	–	–	–	–	–

### Reward systems reveal greater functional connectivity during improvisation

**Fig. 3:**
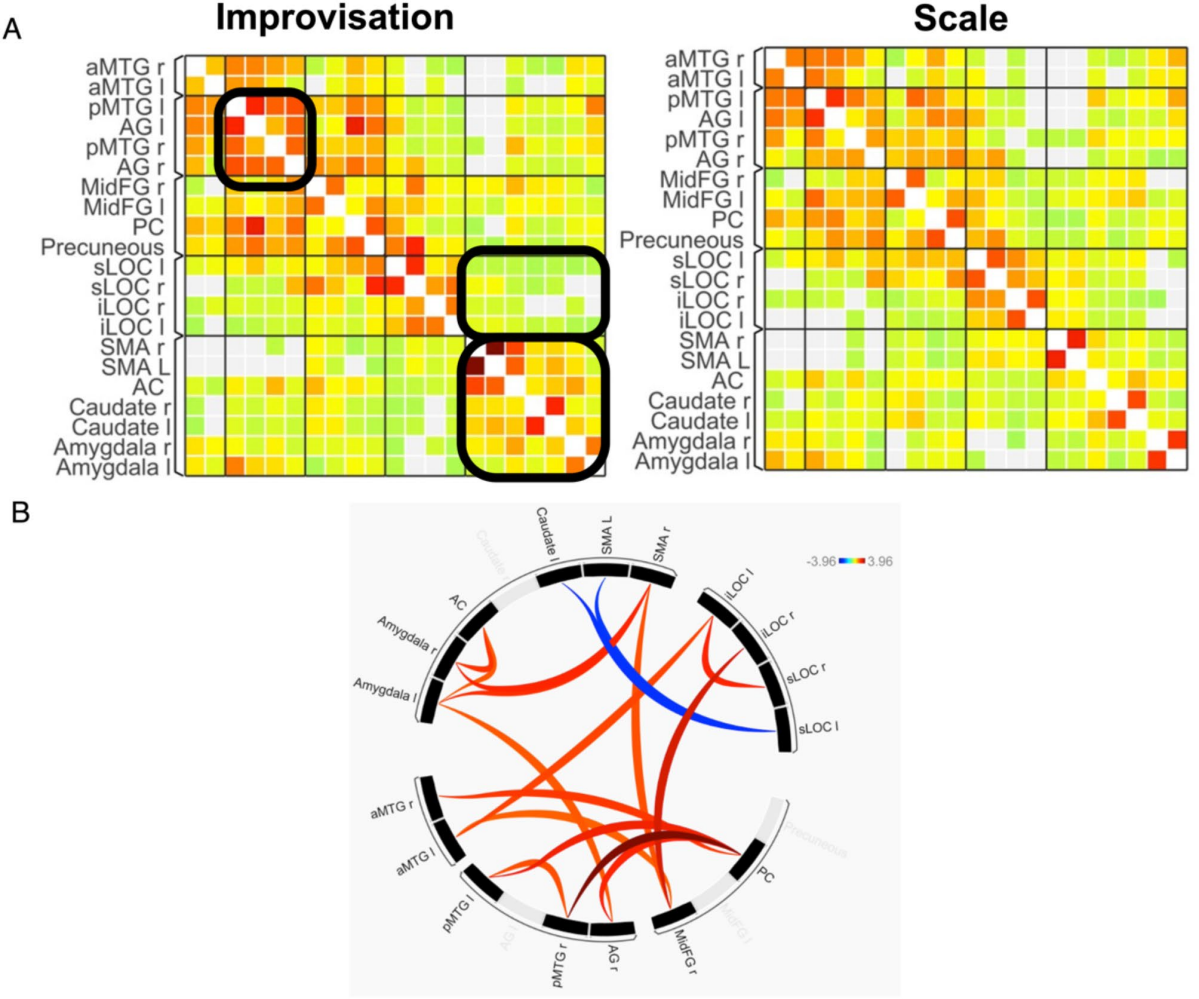
Functional connectivity analysis. Panel (**A**) depicts Within Condition connectivity heat maps. Each map represents a functional connectivity matrix between regions of interest (ROIs). ROIs were those anatomical regions with large clusters from random effects contrast data (see Table 1). Correlated activity is depicted in red-orange, and anti-correlated activity is shown in blue-green, with the intensity bar designating t-scores. Black boxes on the improvisation matrix (left) highlight key functional connectivity differences from the scale condition (right). Panel (**B**) represents a Between Condition connectome depicting which structures have statistically significant correlated activity (in red) or anticorrelated activity (in blue). *a* anterior, *p* posterior, *r* right, *l* left, *s* superior, *i* inferior, *MTG* middle temporal gyrus, *AG* angular gyrus, *FG* frontal gyrus, *SMA* supplementary motor area, *sLOC* superior lateral occipital cortex, *iLOC* inferior lateral occipital cortex, *PC* posterior cingulate cortex, *AC* anterior cingulate cortex. See Supplementary Materials Table 1 for significance values of regions within the three box areas.

Random effects functional connectivity analysis was also performed within conditions to probe for neural network differences between the improvisation and scale control conditions. This analysis used critical neural structures (ROIs) from our contrast analysis and examined whether these structures worked in correlated or anti-correlated activity with each other, as depicted in Fig. 3.

#### Within conditions

Functional connectivity analysis within conditions (within Improv, within Scale) reveals additional nuances (Fig. 3A). A correlation matrix for both conditions was created, and areas of interest were determined via visual inspection of the matrices. Generally, the matrices look similar but with minor differences. Within the improvisation condition, strongly correlated activity was seen between (1) the MTG and AG, (2) the SMA, ACC, Caudate, and Amygdala–namely between areas of the motor, reward, and limbic network, and (3) the precuneus and superior lateral occipital cortex (sLOC). Within the Scale condition, significantly correlated activity was seen between the sLOC and inferior lateral occipital cortex (iLOC)—areas involved in vision and object recognition ^36^. See demarcated boxes in Fig. 3 connectivity matrix.

#### Across conditions

To probe whether connectivity between these targeted structures statistically differed between the improvisation vs. scale control condition, follow-up analyses were performed. Functional connectivity correlation values between pairs of structures were calculated. Post-hoc paired sample *t*-tests compared the correlation values between the improvisation vs. memory control condition, and significant connections were plotted on a connectome graph (Fig. 3B). Improvisation had significantly higher correlated connectivity between the SMA and amygdala, ACC and amygdala, and between the left and right sides of the posterior MTG. Improvisation had stronger anti-correlated activity between the SLOC and SMA L and the SLOC and caudate (Supplementary Materials Table 1). In short, improvisation had significantly stronger connectivity between neural substrates involved in emotion, reward, and motivation (e.g., ACC, amygdala, and caudate), which suggests that improvisation engages reward and emotion areas more strongly than the control condition.

**Table 2:** Analysis of MIDI data obtained for scale (control condition) And improvise (experimental condition) blocks. Data depict mean ± s.d. And the *p*-values from paired sample *t*-tests directly comparing the two conditions. Because the two conditions do not differ on these musical attributes, differences found are likely due to whether the musical output was spontaneously created or pre-learned.

	Scale	Improvise	*p*-value
Number of notes	50.2 ± 6.32	48.8 ± 6.38	0.29
Weighted distribution	65.47 ± 0.19	65.2 ± 0.50	0.08
Melodic complexity	4.14 ± 0.40	4.22 ± 0.33	0.17

Finally, we analyzed the MIDI data from participants’ recorded output to verify that motor movements and musical complexity were controlled between the experimental improvisation and rote control tasks. This signal processing analysis compared the number of notes, weighted distribution (a measure of the range of notes played), and melodic complexity between the scale and improvisation condition. The statistical analysis of piano MIDI performance data by paired sample *t*-tests revealed no significant difference in musical parameters (Table 2). The Scale and Improvise conditions were matched on the average number of notes per block (t(11) = 1.101, *p* = 0.29), the weighted distribution of notes (t(11) = 1.95, *p* = 0.08), and musical complexity (t(11) = − 1.46, *p* = 0.17). Thus, MIDI data analysis reveals that scale and improvisation conditions were matched on low-level motoric demands and musical complexity, controlling for these factors. The main difference between the experimental vs. control conditions was whether the musical output was spontaneously created or pre-planned, in order to ensure that our paradigm tested for the impact of generative creativity.

## Discussion

This neuroimaging experiment provides the first evidence that children possess a nascent, developing creativity network used to perform an artistic generative task like musical improvisation. Both functional connectivity and task-based contrast analysis revealed that improvisation engages reward structures like the caudate, amygdala, and nucleus accumbens more than the control condition, supporting our hypothesis. Contrast data showed that improvisation was associated with deactivation of the amygdala, caudate, and nucleus accumbens as discussed below. Subsequent functional connectivity analysis showed that improvisation resulted in significantly stronger correlated activity between the Amygdala and the SMA, and Amygdala and ACC, and stronger anti-correlated activity between the sLOC and the caudate, signifying that these reward structures work in conjunction to support improvisation in children. Thus, improvisation is associated with more robust engagement of reward structures than memorized tasks, and this may be one of the reasons why children engage in creative activities.

Our fMRI contrast random effects analysis further revealed that children’s creativity is associated primarily with brain deactivation. Children’s creativity network deactivates limbic, parietal, and auditory brain structures like the AG, precuneus, cingulate cortex, postcentral gyrus, and MTG. Because these school-aged children were young, with little to no prior musical training, results suggest that children can be creative without extensive training and are born with a burgeoning, emerging neural creativity network. Results here indicate a nascent neural creativity network. Notably, executive network (EN) structures like the ACC and DLPFC^31^ were deactivated. The EN is utilized for goal-directed tasks reliant on executive functions such as cognitive control, working memory, and cognitive flexibility^37^. Deactivation of EN structures supports our hypothesis that school-aged children did not require cognitive control to perform musical improvisation.

However, while improvisation engages reward structures more strongly than the control condition, the reward valence—positive or negative—reveals a complex story. Amygdala deactivation, as found here, is thought to be related to positive emotions^38^. However, the significance of caudate deactivation, as found here, remains unclear due to conflicting findings in the literature. Caudate activation occurs during goal-directed actions^39^ and anticipation of musical reward^40^, while caudate deactivation is associated with reward-prediction error^41^—the caudate deactivates when subjects miss an expected reward^42^. This complexity is also seen in the nucleus accumbens. Enhanced functional connectivity with the nucleus accumbens is associated with music and positive reward^43,44^, while nucleus accumbens deactivation, as found here, is related to various responses such as passive avoidance of harm^45^, musical anhedonia^46^, and depression^47^. Thus, while the amygdala deactivation during improvisation suggests positive reward and joy, deactivation of the caudate and nucleus accumbens may indicate negative emotional valence, creating a more nuanced story.

There are many reasons why reward valence during musical improvisation may include a mixture of both positive and negative rewards or emotions. It is possible that performing a novel task such as improvisation in an fMRI scanner—which may be intimidating or odd to participants—meant they felt both positive and negative emotions concurrently while improvising. Alternatively, a mixture of positive and negative reward valence may occur due to the sheer cognitive and motoric demands of performing musical creativity in real-time. While previous research in adult professional musicians found that improvisation was associated with positive reward due to the deactivation of the amygdala and hippocampus, reward valence was unclear when related to the deactivation of other limbic structures like the hypothalamus and ventral striatum^10^. Due to the sophisticated processes involved in performing real-time generative musical creativity, it may be more complicated than a simple matter of positive or negative reward. It is important to note that any heightened activation of the limbic/reward system, whether positive or negative in valence, may be more engaging to an individual than a memorized task that leads to no such activity. In the context of a “safe” context such as musical production, perhaps the negative valence emotions experienced during musical play are a vitally important part of the musical experience and part of the process of emotional growth that takes place when individuals engage in creative, artistic behaviors. Furthermore, artistic creativity includes emotional messaging that reflects the entire breadth of the human emotional spectrum. Hence, the most essential feature may be the engagement of the reward system itself, rather than a simple assessment of reward valence as positive or negative. If improvisation engages the reward system, this may explain *why* children gravitate towards creative behaviors.

As mentioned in the introduction, one reason for children’s creativity may be their lack of knowledge and expertise, which may be why there is a raw, playful, and fresh spontaneity to their creativity (see discussion in^48^). Children examined here are the ultimate beginners in terms of extensive musical knowledge and life experience. This experiment shows the neural correlates of the “beginner’s mind”—a term borrowed from meditation. The beginner’s mind is an attitude of openness, eagerness, and lack of preconception. The beginner’s mind is an empty mind ready for anything: “In the beginner’s mind, there are many possibilities, but in the expert’s, there are few”^49^. Children possess highly flexible, pliable minds—characteristics of the beginner’s mind.

In a related vein, perhaps children without extensive training *must* create as they know nothing else; children possess no other schemas or background knowledge to draw upon and, therefore, innovate and create. Perhaps there is a basic neural motivation to generate new ideas as part of being human—an innate desire to be creative. Ideally, this neuroimaging experiment would have been performed in even younger children, such as preschoolers or kindergarteners, to identify innate neural creativity networks. However, due to the practical challenges of conducting an fMRI experiment, focusing on school-aged children (9–11 years old) provided a compromise between logistical considerations and our desire to investigate the earliest neural correlates of creativity. While it can be challenging to perform functional neuroimaging on children (see methods), it is a research pathway that warrants consideration. Adult nonmusicians and young children offer a valuable opportunity to study creativity in the absence of domain-specific expertise (i.e., the beginner’s mind). Additionally, examining children’s creativity across age groups or longitudinally could illuminate their developmental trajectory from childhood to adulthood.

Because child participants possessed little to no musical training, the strong involvement of the AG, precuneus, cingulate cortex, and MTG with improvisation here may represent “native” or intrinsic neural substrates of generative musical creativity. In other words, these structures may be inherent to creativity, as they are not a product of musical expertise or training and are involved even in this early stage of development. The AG, located in the posterior part of the IPL, is a sensory hub, receiving visual input from the occipital lobe, auditory input from the temporal lobe, and somatosensory input from the parietal lobe^50^. As a result, the AG is involved in diverse functions like language and reading comprehension, math and spatial cognition, social cognition, memory retrieval^50^, and integrating sensory modalities. The precuneus is likewise involved in highly integrated tasks, such as visuospatial imagery and self-processing operations, like first-person perspective^51^ or self-referential processing^52^. The cingulate cortex, divided into the ACC, MCC, PCC, and retrosplenial cortex, is also involved in diverse functions like emotion processing, reward-based decision, self-referential processing^53^, and visuospatial orientation^54^. Due to the neural pathways it shares with other brain regions, the cingulate cortex is a connecting hub of emotions, sensations, and actions^54^. Finally, the MTG is likewise involved in multimodal sensory integration^55^. All four structures—the AG, precuneus, cingulate cortex, and MTG—are sensory hubs emphasizing self-referential processing. The fact that they showed the strongest and largest deactivation clusters during improvisation suggests that children are not using self-referential processing or expressing a unique artistic voice. While improvisation can be an artistic expression drawn from a core sense of self-identity in mature artists, this may not be true in young, untrained children. In other words, expertise and domain knowledge may be necessary for certain creative tasks. However, in this experiment focused on beginner musical improvisation as a non-constrained behavior, the AG, precuneus, cingulate cortex, and MTG may be candidates for the neural basis of generative, musical creativity.

As mentioned in the introduction, although neural results vary across creativity experiments due to differences in paradigms, subjects, and equipment^29,30,56^, the mPFC, cingulate cortex, the DLPFC, IFG, dorsal premotor areas, preSMA and supplementary motor areas (SMA), are associated with musical improvisation in adults^28–30,57^. These improvisation brain structures belong to several functional networks, such as the default mode (DMN) and executive networks (EN)^9,58^. Recent research suggests that the salience network, comprised of the bilateral insula and ACC, connects the DMN and EN, identifying ideas generated during improvisation by the DMN and forwarding this information to the EN for higher-order processing like evaluation or revision^59^. Highly creative adults show dense functional connections between the DMN, EN, and salience brain networks^60^. An improviser generates new ideas but selects and evaluates them quickly, utilizing both the DMN and EN^61^. Theoretical models posit that expert improvisers train to gain musical expertise and then store pre-learned motor sequences and musical schemas in long-term memory to select them during improvisation^58,62,63^.

The existing experiments on the neuroscience of musical improvisation test adults, and primarily experts, making it difficult to compare our findings and the existing literature. However, we would like to note that children utilize some of the same neural structures for their creativity as adults, even though children do not have substantial life experience or musical knowledge to draw upon during improvisation. It is possible that the neural creativity network in children found in this experiment (i.e., cingulate cortex, DLPFC, IFG, SMA, MTG, AG) are the roots of creativity networks seen later in adults. For example, several fMRI experiments on adults have specifically implicated the AG, precuneus, and cingulate cortex in musical improvisation^10,11,24,25,64^. These sensory hubs are crucial to domain-general creativity in adults. The AG deactivates during creative tasks^65^, and the precuneus deactivates for divergent thinking tasks such as brainstorming^66^. Perhaps these sensory hubs are present and engaged in musical creativity early in childhood and continue to be refined with development and additional musical training. Furthermore, many neural structures involved in children’s improvisation belong to the DMN (i.e., DLPFC, hippocampus, PCC, precuneus, AG), which plays a crucial role in adult generative creativity. However, it should be emphasized that DMN regions in children this age are sparsely connected and only integrate into a cohesive, functionally interconnected network later in development^67^. Because children do not yet have functional networks (such as the DMN), we again emphasize that it is not easy to compare our experimental findings directly to existing research in adults.

Children’s neural substrates for creativity diverge from what has been seen experimentally in adult experts. For example, adult jazz experts showed a distinctive widespread deactivation of the DLPFC when improvising^10^, which was associated with a flow state. Children showed some DLPFC deactivation but to a lesser extent than highly trained adult jazz musicians. One major limitation of this experiment is that there is a clear difference between adult jazz experts performing sophisticated musical tasks^10^ and children performing a limited, beginner’s paradigm. In this experiment, it is plausible that children experienced less of a flow state due to the constraints of the beginner’s paradigm. Thus, this experiment cannot directly disentangle the impact of expertise vs. development. Because this is the first functional neuroimaging experiment on creativity in children, more research is needed to unravel these components. Another limitation of our experiment is that while the control and improvisation paradigms were matched in terms of musical complexity and motor movement as shown through our MIDI data analysis, it is possible that the control condition was “boring” to the children, lacking some of the musical and auditory interest of the improvisation condition. If so, this may be why the improvisation condition engaged the limbic and reward structures. We would, however, argue that while playing a pentatonic scale might seem repetitive or unmusical, it was rendered musical and pleasant because of the provided backtrack (see Supplementary Materials). The backtrack provided musical structure and “groove” even to the control condition. Moreover, we believe this is an ecologically valid paradigm as improvising upon the pentatonic scale is a common beginner improvisation exercise^68–70^. However, future experiments may want to explore using simple rehearsed musical pieces as the control condition (ex. “Twinkle Twinkle Little Star” or “Hot Cross Buns”). Using a rehearsed musical piece was beyond the scope of this experiment, given our time limitations in training child participants to enter the fMRI scanner, but it is something that could be considered in future experiments.

Considering the essential importance of creativity to human innovation, invention, and evolution, we believe neuroimaging experiments on creativity provide critical insights into these complex processes. Our findings show that musical improvisation in children engages reward structures of the brain more than rote performance. These reward structures are part of a developing, nascent neural creativity network that is present even without extensive training or expertise. This experiment is a starting point for additional investigations of creativity’s biological and neural substrates. Further studies should examine the development of creative brain mechanisms with age to connect the large gap between naive children and expert adults. In addition, further investigation is needed of neural mechanisms of creativity in other domains beyond music, including other art forms, but also those types of creativity viewed as more prosaic.

## Methods

### Participants

Experimental participants (*n* = 12, 4 females and 8 males) were 9–11-year-old children with no cognitive or neurological impairments and no ferromagnetic indwelling implants. Participants were not musically trained, although some had small amounts of music lessons in a variety of settings and formats (school music classes vs. privately at home, group lessons vs. individual lessons) as well as instruments (mean years of formal musical training = 2.43 years, range = 0–7 years). Children were not practicing instruments extensively (mean number of hours/week practicing = 2.58 h).

Before their lab visit to UCSF, all participants were sent links to sound files (http://cibsr.stanford.edu/GettingReady/HomePreparation.html created by the Stanford Interdisciplinary Brain Sciences Research center) of fMRI machines to acquaint them with the scanning environment and loud sounds the scanner would create. The research protocol was approved by the University of California, San Francisco (UCSF) Institutional Review Board and carried out per approved guidelines. Informed consent was given by parents or legal guardians for each participant. All methods were performed in accordance with the relevant guidelines and regulations. Participants were compensated for their time, given a picture of their brain as a souvenir, and allowed to pick two toys out of the lab “treasure chest” as a reward for participation.

### Stimuli/musical paradigm

A block design paradigm assessed neural correlates associated with improvisation instead of rote musical activity in children. The Scale control condition assessed brain activity during a rote musical task. The experimental Improvise condition assessed brain activity when children engaged in a generative, creative musical activity. In Scale, participants were told to play the pentatonic scale (the five black note keys) up and down to the provided beat with their right hand only. In Improvise, participants were told to play those same notes of the pentatonic scale to the beat with the right hand only, but playing the keys in whatever order desired, thus improvising short musical melodies (Fig. 1).

The tempo of the accompaniment backtrack featuring supporting harmonies and rhythms (created in GarageBand, Apple, Inc.) was 99 beats per minute. 20-s rest blocks separated five 34-s blocks of each task for a total time of 9 min (each block consisted of 12 measures). One run consisted of a randomized presentation of these ten blocks, followed by a brief break where participants remained in the scanner. The second run was a repeat of Run 1 to gather more data. Stimuli were presented using E-Prime 2.0.

### Pre-scanning training

After consent, in a room down the hall from the scanner, participants completed a “Statue Game” where they lay supine and balanced a Lego™ on their forehead, adding a new Lego™ every 30 s until 2 min. The Statue Game taught participants the physical stillness required to achieve artifact-free scanning images. Participants were also shown a picture that depicted how body stillness achieved a good brain picture, while movement would lead to a blurry brain picture. After the statue game, participants received training on the musical task that would be performed in the fMRI machine. Participants used a 25-key Oxygen USB MIDI keyboard (M-Audio, Los Angeles, CA) hooked to a Lenovo Thinkpad laptop running a free version of Ableton-Live (Ableton, Berlin, Germany). Participants practiced the Control (Scale) block and Experimental (Improvise Block) to practice playing a keyboard and the pentatonic notes above the given backtrack. For many participants, this was their first time playing a keyboard or engaging in musical activities. The first author, an accomplished pianist, coached participants on how to play the keyboard comfortably. This task was easily accessible and easy to perform without prior musical experience. Moreover, given the harmonies in the backtrack and the use of the pentatonic scale for the melody, the musical output produced by the children always sounded appropriate. This carefully designed “beginner’s paradigm,” in which it was impossible to make a “mistake,” aided the kids’ enthusiasm for the experiment, reducing any inhibitions or fears about playing piano.

#### Procedure

During scanning, subjects played a custom-built, non-ferromagnetic piano keyboard (MagDesign, Redwood, CA) with thirty-five full-size plastic piano keys. The keyboard had Musical Instrument Digital Interface (MIDI) output, which was sent to a Macintosh Macbook Pro laptop computer running the Logic Express 9 sequencing environment (Apple Inc., Cupertino, CA). The MIDI input triggered high-quality piano samples using the Logic EXS24 sampler plug-in. Piano sound output was routed back to the subject via the Mackie Onyx 820i audio mixer (Loud Technologies, Woodinville, WA) to the over-ear pneumatic headphones (Siemens). Participants lay supine in the scanner with the piano keyboard on their lap while their knees were elevated with a bolster. A double mirror attached to the head coil allowed participants to see the keyboard and their fingers in the scanner. Structural scanning was acquired before functional scanning. Participants were monitored visually from the control room to ensure they did not move the head, trunk, limbs, or feet during scanning. In addition to the pneumatic headphones, subjects wore hearing protection to minimize background scanner noise. The headphone volume was set to a comfortable listening level that could easily be heard over the background scanner noise. The experimenter in the control room could hear the subject and audio stimuli via an M-Audio Studiophile AV40 free-field monitor (M-Audio, Cumberland, RI). Scanning breaks were given as needed to ensure children were comfortable. Parents oftentimes waited in the control room as well.

### fMRI scanning parameters

Blood oxygen level-dependent imaging (BOLD) data were acquired using a 3-Tesla whole-body scanner (Siemens Prisma Fit) using a 64-channel head coil. The following scanning parameters were used: TR = 1000 ms, TE = 32.8 ms, Flip angle = 45 degrees, Field of View = 211 × 211 mm, 66 slices, each voxel = 2.2 mm × 2.2 mm × 2.2 mm. Initial dummy scans were acquired during the establishment of equilibrium and discarded in the data analysis. For each subject, 540 volumes were acquired in each run (across the Scale, Improvise, and Rest Blocks); two runs were acquired for each subject as described above.

### fMRI contrast analysis

Standard preprocessing steps were completed in SPM12, including realignment, coregistration, segmentation, normalization, and smoothing with an 8 mm FWHM kernel. A first-level general linear model (fixed effects analysis) was estimated for each subject using three regressors (e.g., one for rest, Scale, and Improvise ). Each regressor was convolved with a standard hemodynamic response function. Design matrices also included covariates of non-interest, which consisted of motion parameters calculated during the realignment stage and mean signal intensity for the run. All contrasts (i.e., Improvise-Rest, Scale-Rest, Rest-Improvise, Rest-Scale, Improvise-Scale, Scale-Improvise) were estimated for each subject.

Contrasts were then entered into a second-level, random-effects model using a one-sample *t*-test. Random-effects analyses consider inter-subject variability and can be generalized to a broader population. Inclusive masking (default *p* < 0.05) was used to acquire true activations and deactivations: Improvise-Scale was masked by Improvise-Rest, and Scale-Improvise was masked by Rest-Improvise. Contrasts were thresholded at an uncorrected *p*-value of 0.005 for true activations and an uncorrected p-value of 0.001 for true deactivations. The minimum cluster size was 10 voxels. SPM Anatomy Toolbox (Forschungszentrum Jülich GmbH)^71^ was used to create *minima* and *maxima* tables for anatomical locations of voxel clusters. Anatomical results of these *minima/maxima* tables were then confirmed using the Automated Anatomical Labeling Toolbox^72^.

An effect size comparison was conducted to measure contrast estimates between experimental and control conditions using the MNI coordinates of critical regions in the *minima* and *maxima* tables. Effect size data was calculated using the “Contrast Estimate” function in SPM12.

### Functional connectivity data analysis

Results included in this manuscript come from analyses performed using CONN^73^ release 22.a^74^ and SPM12.

Functional and anatomical data were preprocessed using a flexible preprocessing pipeline^75^, which included realignment with correction of susceptibility distortion interactions, outlier detection, direct segmentation and MNI-space normalization, and smoothing. Functional data were realigned using SPM realign and unwarp procedure^76^, where all scans were coregistered to a reference image (first scan of the first session) using a least squares approach and a 6 parameter (rigid body) transformation^77^, and resampled using b-spline interpolation to correct for motion and magnetic susceptibility interactions. Potential outlier scans were identified using ART^78^ as acquisitions with framewise displacement above 0.9 mm or global BOLD signal changes above 5 standard deviations^79,80^, and a reference BOLD image was computed for each subject by averaging all scans excluding outliers. Functional and anatomical data were normalized into standard MNI space, segmented into grey matter, white matter, and CSF tissue classes, and resampled to 2 mm isotropic voxels following a direct normalization procedure^81,82^ using SPM unified segmentation and normalization algorithm^83,84^ with the default IXI-549 tissue probability map template. Finally, functional data were smoothed using spatial convolution with a Gaussian kernel of 8 mm full width half maximum (FWHM).

### Denoising

In addition, functional data were denoised using a standard denoising pipeline^85^, including the regression of potential confounding effects characterized by white matter time series (5 CompCor noise components), CSF time series (5 CompCor noise components), motion parameters and their first order derivatives (12 factors)^86^, outlier scans (below 176 factors)^79^, session and task effects and their first order derivatives (6 factors), and linear trends (2 factors) within each functional run, followed by high-pass frequency filtering of the BOLD timeseries^87^ above 0.003 Hz. CompCor^88,89^ noise components within white matter and CSF were estimated by computing the average BOLD signal and the largest principal components orthogonal to the BOLD average, motion parameters, and outlier scans within each subject’s eroded segmentation masks. From the number of noise terms included in this denoising strategy, the effective degrees of freedom of the BOLD signal after denoising were estimated to range from 753.5 to 1007.9 (average 911.8) across all subjects^80^.

### First-level analysis

ROI-to-ROI connectivity (RRC) matrices were estimated, characterizing the functional connectivity between each pair of regions among 21 Harvard-Oxford atlas ROIs^90^. Functional connectivity strength was represented by Fisher-transformed bivariate correlation coefficients from a general linear model (weighted-GLM^91^), estimated separately for each pair of ROIs, characterizing the association between their BOLD signal time series. Individual scans were weighted by a boxcar signal characterizing each individual task or experimental condition convolved with an SPM canonical hemodynamic response function and rectified.

Group-level analyses were performed using a General Linear Model (GLM^92^). For each individual connection a separate GLM was estimated, with first-level connectivity measures at this connection as dependent variables (one independent sample per subject and one measurement per task or experimental condition, if applicable), and groups or other subject-level identifiers as independent variables. Connection-level hypotheses were evaluated using multivariate parametric statistics with random-effects across subjects and sample covariance estimation across multiple measurements. Inferences were performed at the level of individual clusters (groups of similar connections). Cluster-level inferences were based on parametric statistics within- and between- each pair of networks (Functional Network Connectivity^93^), with networks identified using a complete-linkage hierarchical clustering procedure^94^ based on ROI-to-ROI anatomical proximity and functional similarity metrics^95^. Results were thresholded using a combination of a *p* < 0.05 connection-level threshold and a familywise corrected p-FDR < 0.05 cluster-level threshold^96^.

### MIDI data analysis

We applied signal processing methods to analyze the MIDI piano output obtained during fMRI scanning. This analysis aimed to quantitatively evaluate that the scale and improvise conditions were matched on specific musical parameters: *number of notes played*, *weighted distribution of notes*, and *musical complexity*. Each subject’s total number of notes played for the MIDI piano data was tabulated for each condition. If participants played every note perfectly, each block (Scale and Improvise) should have 49 notes. A weighted distribution of notes was calculated as a quantitative measure that reflected the absolute range of notes and the distribution of keyboard notes played (and, to a limited extent, the physical movements required). The weighted distribution was computed by taking the mean of the MIDI pitch value of all notes played, which was weighted by the number of times each individual note was played. Since participants played the pentatonic notes C^#^4–A^#^4 on the piano keyboard, the weighted distribution should be 65.6 (the average value of those notes). Finally, *musical complexity* was calculated using the MIDI Toolbox^97^. The melodic complexity rating weighs factors such as tonal stability, accents, pitch skips, rhythmic variability, and melodic contours^97–99^. Melodic complexity scores range from 0 to 10, where higher scores indicate more complex tunes. For all three parameters, paired *t*-tests were used to compare piano output during Scale and Improvise conditions (Table 2) as a control to ensure that the two conditions matched regarding low-level motoric output.

## Electronic supplementary material

Below is the link to the electronic supplementary material.

Supplementary Material 1

Supplementary Material 2

Supplementary Material 3

## Data Availability

For data sharing requests, please correspond with the lead contact Karen Barrett (Karen.Barrett@ucsf.edu). Data will also be available on Dryad.
