# Supplementary material for: Children engage neural reward structures for creative musical improvisation
Source: Sci Rep. 2025 Apr 10;15:11346. doi: 10.1038/s41598-025-95619-1 (PMC11986006; doi:10.1038/s41598-025-95619-1)
Supplement: Supplementary file 1 — Supplementary Material 1 [file 41598_2025_95619_MOESM1_ESM.docx]

Supplementary Materials

| **Neural Structures Involved** | **Significance** | **Improvisation**  **Mean +/- SD** | **Scale**  **Mean + SD** |
| --- | --- | --- | --- |
| ACC * Amygdala R | t(11) =-2.567, p < 0.05 | 0.318 +/- 0.097 | 0.248 +/- 0.114 |
| ACC *Amygdala L | t(11) = -2.268 , p < 0.05 | 0.312 +/- 0.139 | 0.221 +/- 0.109 |
| SMA R * Amygdala R | t(11) = -2.699, p < 0.05 | 0.200 +/- 0.093 | 0.119 +/- 0.010 |
| SMA R * Amygdala L | t(11) = -2.601, p < 0.05 | 0.163 +/- 0.1332 | 0.083 +/- 0.092 |
| Posterior MTG L * posterior MTG R | t(11) = -2.341, p <0.05 | 0.697 +/- 0.160 | 0.626 +/- 0.158 |
| sLOCL * SMA L | t(11) = 2.515, p < 0.05 | 0.128 +/- 0.132 | 0.208 +/- 0.092 |
| sLOCL * Caudate L | t(11) = 2.786, p < 0.05 | 0.101 +/- 0.120 | 0.191 +/- 0.096 |

**Supplementary Table 1. Between Condition Functional Connectivity Paired Sample T-tests of functional connectivity correlation values.** Significant connections between regions were determined by Conn Toolbox. To complete this table, correlation values were calculated between different neural structures and then paired sample-t tests were performed comparing the Improvisation vs Scale control condition correlation values. First column on the left denotes the two regions that are functionally connected. The significance value column lists statistics formatted as [t(df) = t stat, p-FDR value]. The Last two columns on the right list the mean correlation value +/- SD for the Improvisation condition and the Scale Control condition. Red text denotes significant correlated activity; blue text denotes significant anti-correlated activity. Abbreviations: ACC = anterior cingulate cortex, SMA = supplementary motor area, L = Left, R= Right, MTG = Middle temporal Gyrus, sLOC= superior lateral occipital cortex.


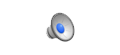

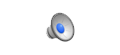


**Sample Improvisation and Control Sound Files (MusicS1 and Music S2).** These .m4a sound files capture sample output by a randomly chosen participant for the scale condition (Music S1) versus the improvised (Music S2) condition. In addition to the melodic output, you can hear that the same instrumental backtrack is used for both conditions. Verbal cues gave the instructions.
